# Prevalence of Alzheimer’s Pathologic Endophenotypes in Asymptomatic and Mildly Impaired First-Degree Relatives

**DOI:** 10.1371/journal.pone.0060747

**Published:** 2013-04-17

**Authors:** Erika J. Lampert, Kingshuk Roy Choudhury, Christopher A. Hostage, Jeffrey R. Petrella, P. Murali Doraiswamy, the Alzheimer’s Disease Neuroimaging Initiative

**Affiliations:** 1 Department of Psychiatry, Duke University Medical Center, Durham, North Carolina, United States of America; 2 Department of Radiology, Duke University Medical Center, Durham, North Carolina, United States of America; 3 The Duke Institute for Brain Sciences, Duke University Medical Center, Durham, North Carolina, United States of America; Nathan Kline Institute and New York University School of Medicine, United States of America

## Abstract

**Objective:**

A positive family history (FH) is a risk factor for late-onset Alzheimer’s disease (AD). Our aim was to examine the effects of FH on pathological and neuronal loss biomarkers across the cognitive spectrum.

**Design:**

Cross-sectional analyses of data from a national biomarker study.

**Setting:**

The Alzheimer’s Disease Neuroimaging Initiative national study.

**Patients:**

257 subjects (ages 55–89), divided into cognitively normal (CN), mild cognitive impairment (MCI), and AD groups, with CSF and FH data.

**Outcome Measures:**

Cerebrospinal fluid (CSF) Aβ42, tau, and tau/Aβ42 ratio, MRI-measured hippocampal volumes.

**Statistics:**

Univariate and multivariate analyses.

**Results:**

In MCI, CSF Aβ42 was lower (p = .005), t-tau was higher (p = 0.02) and t-tau/Aβ42 ratio was higher (p = 0.002) in FH+ than FH− subjects. A significant residual effect of FH on pathologic markers in MCI remained after adjusting for ApoE4 (p<0.05). Among CN, 47% of FH+ exhibited “pathologic signature of AD” (CSF t-tau/Aβ42 ratio >0.39) versus 21% of FH− controls (p = 0.03). The FH effect was not significant in AD subjects. Hippocampal and intracranial volumes did not differ between FH+ and FH− subjects in any group.

**Conclusions:**

A positive family history of late-onset AD is associated with a higher prevalence of an abnormal cerebral beta-amyloid and tau protein phenotype in MCI. The unexplained genetic heritability in family history is about the half the size of the ApoE4 effect. Longitudinal studies are warranted to more definitively examine this issue.

## Introduction

It is estimated that more than 25 million people worldwide have Alzheimer’s disease (AD), with these numbers expected to triple by the year 2050 [1]. Familial early-onset AD is well recognized as an entity [2] but accounts for only about 2–3% of AD cases. FH is also a significant risk factor for, and predictor of late-onset AD [2]–[4] and studies suggests a 2 to 4-fold greater risk for AD in such individuals with a first-degree relative who has developed late-onset AD. Common gene polymorphisms (e.g. the ε4 variant of the APOE gene) account for about 50% of the heritability of late-onset AD [2] and despite recent genetic findings of new candidate genes, there is still a significant unexplained heritability. If one assumes that the average person with AD has 3 living first-degree relatives (1 sibling, 2 children), then there would be some 75 million worldwide with a positive FH of AD. Subjects with a positive FH often participate in research studies as controls, thus making it particularly important to understand how FH affects biomarker phenotype.

While previous multisite research has shown associations between AD biomarkers and specific genetic variations (such as APOE, APP, PSEN1, and PSEN2) [5]–[11], in most research studies and in routine practice, clinicians usually rely on a simple “yes/no” of self-reported FH status. Prior studies of the effects of FH status on biomarkers have shown effects on PET glucose metabolism, hippocampal volumes and amyloid markers [11]–[18]. If true, these findings have great significance since subjects with a positive FH are routinely enrolled in diagnostic and prognostic biomarker studies as “normal controls” and their inclusion might affect the accuracy of biomarker cut-points established for discriminating AD from controls.

The Alzheimer’s Disease Neuroimaging Initiative (ADNI) is considered one of the more successful biomarker research studies and has been widely analyzed and reported [19]–[22]. Its data forms the basis, in part, for newly proposed research diagnostic criteria for preclinical AD as well as MCI due to Alzheimer’s [23]–[24]. A positive family history was not an exclusion for controls or MCI in ADNI nor its subsequent studies ADNI-GO and ADNI-2. Most ADNI subjects were recruited from the community using advertisements and referrals in a manner similar to that used for most therapeutic and biomarker trials in many disorders.

The aims of our study were to systematically assess how subjects with a FH of AD differed from those without a FH in demographics, cognition, as well as neuronal and pathologic biomarker profiles, at the time of enrollment into a national biomarker study. Another aim was to examine whether the effect of FH was solely due to ApoE4 status. We also examined whether the effects of FH status on biomarkers differed across the spectrum of cognitively normal (CN) to MCI to mild AD to test the timing of such effects in relation to development of cognitive symptoms and clinical dementia.

## Materials and Methods

### Subjects

Data used in the preparation of this article were obtained from the Alzheimer’s Disease Neuroimaging Initiative (ADNI) database (adni.loni.ucla.edu) [19]. The primary goal of ADNI has been to test whether serial magnetic resonance imaging (MRI), positron emission tomography (PET), other biological markers, and clinical and neuropsychological assessment can be combined to measure the progression of mild cognitive impairment (MCI) and early Alzheimer’s disease (AD). ADNI subjects have been recruited from over 50 sites across the U.S. and Canada. The initial goal of ADNI was to recruit 800 adults, ages 55 to 90, to participate in the research and have approximately 200 cognitively normal older individuals be followed for 3 years, 400 people with MCI be followed for 3 years and 200 people with early AD be followed for 2 years. For up-to-date information, see www.adni-info.org. To read about the subject eligibility criteria for the ADNI database refer to the ADNI-1 Procedures manual [20]–[21].

#### Ethics

ADNI was approved by the IRBs of all participating sites including Duke University. All subjects and if applicable, their legal representatives, gave written informed consent prior to the collection of clinical, genetic and imaging data.

Subjects selected for analysis were required to have information for all of the following available: age, gender, and family history of Alzheimer Disease; an Alzheimer’s Disease Assessment Scale Cognitive Subscale score (ADAS-Cog) and a Mini-Mental State Examination (MMSE) score obtained at the initial visit; *APOE* genotyping results; initial-visit 1.5 T MRI scans which were analyzed for ADNI by FreeSurfer software, version 4.4; a measure of estimated intracranial volume derived from any MR scan; and baseline values for CSF amyloid-beta 42 (Aβ42), total-tau (t-tau), and phosphorylated tau-181p (p-tau). We chose CSF Aβ42, t-tau, p-tau (a sensitive marker of amyloid and tangles) and MRI-hippocampal volume (a marker of hippocampal neuronal loss). We also looked at MMSE and ADAS, the two measures most often used in practice and clinical trials, respectively. We did not examine other measures to limit the number of comparisons. Not all subjects underwent CSF studies in ADNI and hence our sample was restricted to those that had CSF data. At the time of our data gathering, April 1st 2012, a total of 257 subjects in ADNI-1 met criteria for inclusion in the study.

### Family History and Clinical Diagnosis

ADNI collected FH data using an interview with the subject and their study partner about the presence of Alzheimer’s disease in their parents or siblings and the site checked yes or no to parents and each individual sibling. The source of information was usually study partner for memory-impaired subjects and the subjects themselves for controls. A positive family history (FH+) was defined as having a parent or sibling–living or deceased–who had been diagnosed with AD. A negative FH (FH−) thus consisted of having no parents or siblings with a history of AD. Study participants with uncertain family history status were excluded from the analysis. 247 participants were excluded from the original ADNI database for having unknown or incomplete family history data before the addition of other variables.

### Cerebrospinal Fluid Collection and Assays

CSF samples were obtained by lumbar puncture and examined for t-tau, p-tau181P, and Aβ42 as described previously [21]–[22]. More detailed protocols can be found on the ADNI website [20]. CSF proteins were used as continuous variables in the logistic regression and cut-off values for CSF signatures for AD (CSF t-tau/Aβ42>0.39) were derived from a published autopsy verified correlative study of ADNI subjects [22].

### APOE Genotyping

Genotyping of all subjects for *APOE* allele status was performed using DNA extracted from peripheral blood cells (ADNI-1 Procedures).

### MR Imaging Acquisition

ADNI used 1.5T MP-RAGE T1-weighted MR images. All scans were performed using a standardized protocol specifically developed for ADNI, and which was tailored for use with each make and model of scanner used at the different data collection sites. More detailed information for the specific MR acquisition protocols and quality control methods for each type of scanner used can be found at http://adni.loni.ucla.edu [20].

### MR Volumetric Methods

Hippocampal volumes (HV) were derived from volumetric segmentation of subject MR scans, which were performed with the Freesurfer image analysis suit. Freesurfer morphometric procedures have been demonstrated to show good test-retest reliability across scanner manufacturers and across field strength [25]. Intra-cranial volumes (ICV) were derived from MRI scans (see adni.loni.ucla.edu and refer to the detailed ADNI 1 MRI Protocols for sequences and processing steps).

### Statistical Analysis

**Figure 1:**
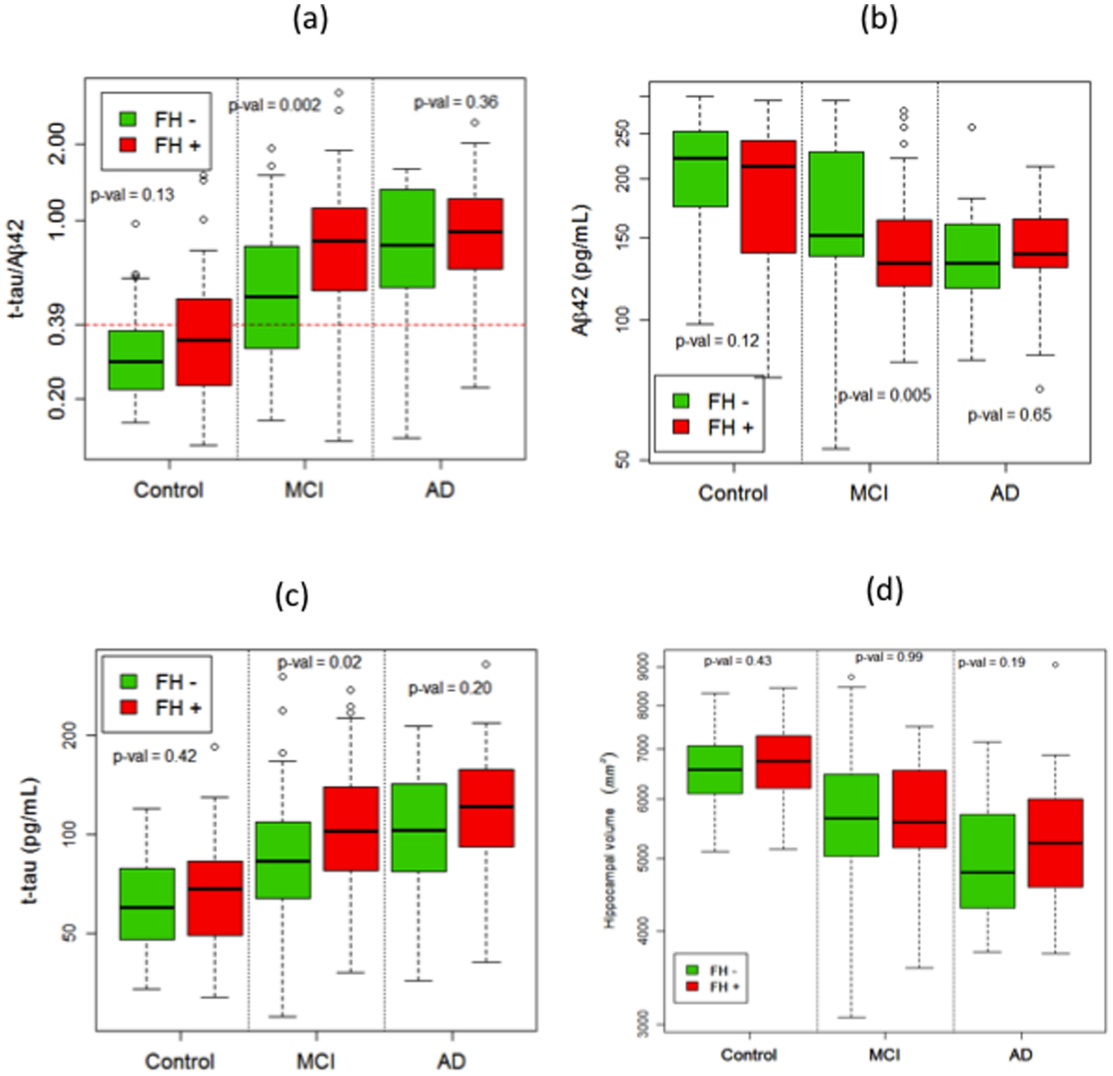
CSF Biomarkers and Hippocampal Volume in CN, MCI, and AD by FH status. a–d. Comparative boxplots of biomarker distribution and hippocampal volume in (mm3) as a function of diagnosis (on x-axis) and family history (present = +, absent = −): (a)TAU/Aβ42 (b)Aβ42 (c)TAU. The y-axis of each plot is on a logarithmic scale to transform the biomarker or hippocampal volume distribution to approximately Gaussian after adjusting for differences due to diagnosis and family history. P-values give the significance of a test of equality between the mean levels of biomarker or hippocampal volume (on the logarithmic scale) in FH+ and FH− groups, within each diagnosis category, using a two-tailed two sample t-test. In (a), the dashed horizontal line at 0.39 denotes the standard threshold value for diagnosis of AD.

**Figure 2:**
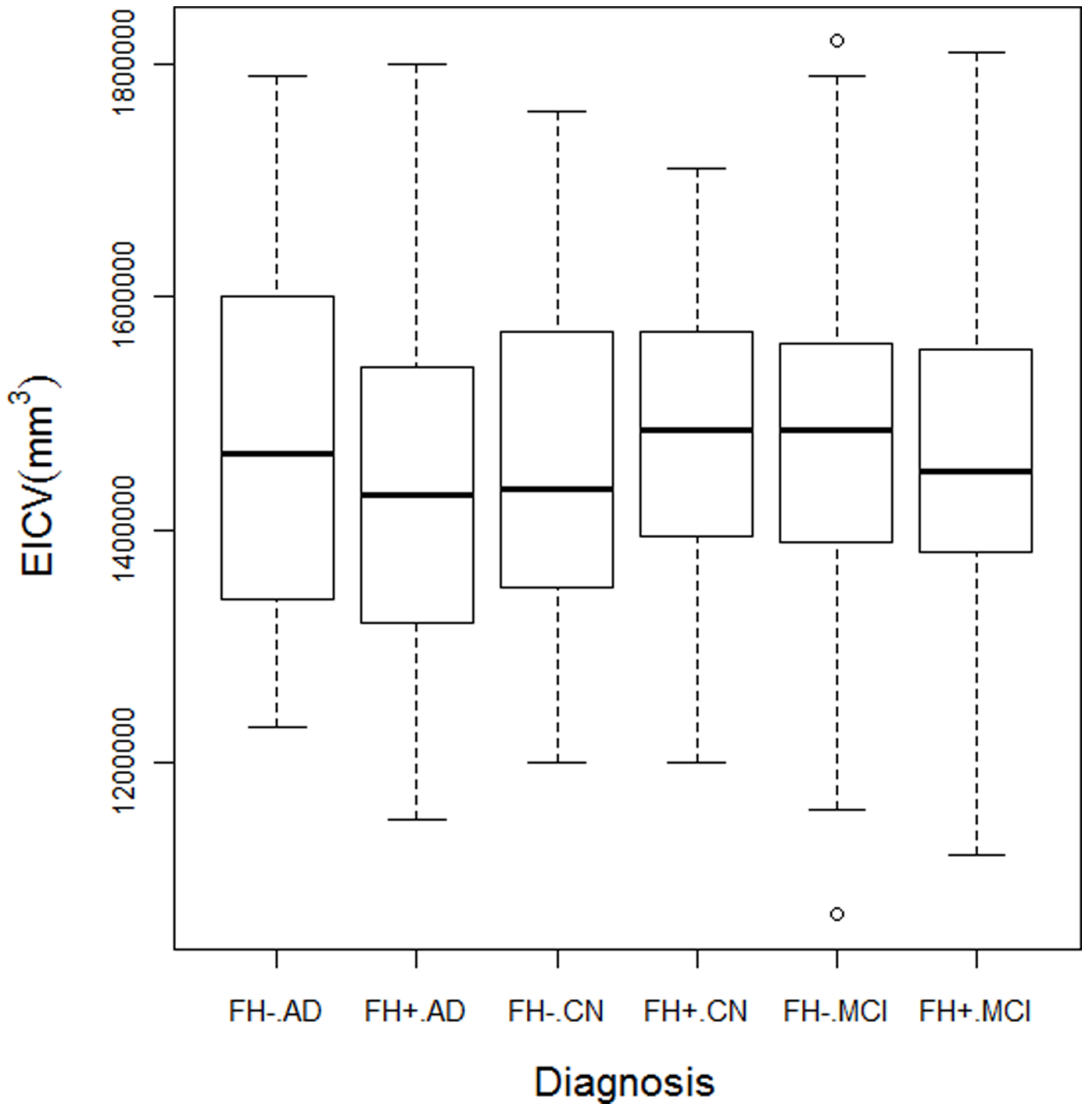
Intracranial Volumes by FH status. Figure shows that ICV (intracranial volume) did not significantly vary by FH status within diagnostic groups. The p-values are from a model covarying diagnosis and FH status only but similar findings were noted in a multivariate model covarying for age, gender, and ApoE4 also. Only gender had an effect on ICV (data not shown).

We first ran unadjusted t-tests and chi-square analyses comparing demographics and biomarker data (CSF Aβ42, t-tau, ptau181p, and hippocampal volume, HV) between FH+ and FH− subjects within each diagnostic group. Biomarker data were log-transformed to normalize their distributions but results were essentially similar before and after log transformation. Table 1 shows demographics and Figures 1–2 show unadjusted biomarker data. We then ran a multivariate linear model, with age, baseline MMSE, gender, and ApoE4 as predictors to examine effect of FH on tau/Aβ42 and HV within each diagnostic group above and beyond the effects of ApoE4 (Tables 2 and 3). We also examined the effect of FH status on intracranial volume (ICV) in each diagnostic group using multivariate linear models and also adjusted for ICV in models examining the effect of FH on HV. With chi-square, we tested the hypothesis that a positive FH status would be associated with a greater proportion of CN and MCI subjects with a CSF pathological signature of AD (CSF tau/Aβ42>0.39). Finally, ROC curves were generated to examine whether the specificity and sensitivity of CSF Tau/Aβ42 ratio for distinguishing AD from FH− CN differed from that for FH+ CN. We also ran alternative models adjusting for education and without adjusting for MMSE.

**Table 1:** Characteristics of ADNI Study Sample.

	CONTROL			MCI			AD		
	FH+	FH−	p-value	FH+	FH−	p-value	FH+	FH−	p-value
N	36	38		56	65		37	25	
Age	74.08	75.13	0.51	73.35	74.33	0.95	72.95	75.28	0.51
Gender ratio (f/m)	1	1	1	0.93	0.48	0.11	0.94	0.73	0.81
Education	15.69	15.63	0.93	15.86	15.64	0.85	15.89	14.58	0.22
% ApoE4+	41.7%	21.1%	.001*	67.85%	43.07%	.005*	75.68%	57.69%	.073
MMSE	28.86	29.34	0.22	26.91	27.03	0.48	23.59	23.46	0.3
ADAScog11	6.55	5.62	0.1864	11.44	11.36	0.7136	18.19	17.6	0.5958

Note: P-values are from two-sample t-tests or chi-square tests for comparisons of FH+ versus FH− subjects within each diagnostic group. The p-values are not for across group comparisons.

**Table 2:** Effect of FH on t-tau/Aβ42 ratios after covarying for ApoE4.

CN	Estimate	Std. Error	p-value
(Intercept)	0.1046	0.0933	0.2660
FH+	0.0948	0.0585	0.1095
APOE4+	0.1778	0.0653	**0.0082**
Age	0.0033	0.0052	0.5254
Gender	0.0407	0.0582	0.4873
MMSE	0.0690	0.0281	**0.0166**
**MCI**	**Estimate**	**Std. Error**	**p-value**
(Intercept)	−0.8461	0.1187	0.0000
FH+	0.2342	0.1103	**0.0360**
APOE4+	0.4843	0.1126	**0.0000**
Age	0.0016	0.0081	0.8399
Gender	−0.0034	0.1155	0.9769
MMSE	−0.0529	0.0290	0.0706
**AD**	**Estimate**	**Std. Error**	**p-value**
(Intercept)	−0.4667	0.2254	0.0430
FH+	0.0750	0.1482	0.6146
APOE4+	0.2499	0.1616	0.1276
Age	−0.0094	0.0099	0.3463
Gender	−0.1203	0.1430	0.4038
MMSE	−0.0259	0.0376	0.4930

Table shows results of multivariate model of the effect of FH, after covarying for ApoE4, in Controls, MCI and AD. The effect of FH was significant in MCI.

**Table 3:** Effect of FH on HV after covarying for ApoE4.

Controls	Estimate	Std. Error	p-value
(Intercept)	8.8286	0.0452	0.0000
FH+	0.0082	0.0283	0.7726
APOE4+	0.0054	0.0316	0.8648
Age	−0.0064	0.0025	**0.0129**
Gender	−0.0062	0.0282	0.8278
MMSE	−0.0120	0.0136	0.3810
**MCI**	**Estimate**	**Std. Error**	**p-value**
(Intercept)	8.6172	0.0335	0.0000
FH+	0.0234	0.0312	0.4552
APOE4+	−0.0746	0.0318	**0.0206**
Age	−0.0084	0.0023	**0.0003**
Gender	0.0687	0.0327	**0.0379**
MMSE	0.0266	0.0082	**0.0016**
**AD**	**Estimate**	**Std. Error**	**p-value**
(Intercept)	8.5258	0.0607	0.0000
FH+	0.0485	0.0401	0.2317
APOE4+	−0.0345	0.0444	0.4407
Age	−0.0092	0.0027	**0.0013**
Gender	0.1130	0.0390	**0.0053**
MMSE	0.0153	0.0102	**0.1393**

Table shows results of multivariate model of the effect of FH on hippocampal volume, after covarying for ApoE4, in Controls, MCI and AD. Age, gender and cognitive status were more closely linked to HV in this model. There was no effect of FH on HV. Intracranial volume did not differ by FH status but was influenced strongly by gender.

## Results

The overall, prevalence of FH+ in subjects who volunteered for CSF in ADNI-1 (50.4%) was slightly higher than the prevalence of FH+ among all ADNI-1 subjects with FH data (42%). The prevalence of a FH+ status did not differ significantly between diagnostic groups (p = 0.36). Within each diagnostic group, there were no differences in key demographic or cognitive baseline characteristics by FH status (Table 1).

### Effect of FH before Adjusting for ApoE4

Figure 1a–d depicts the unadjusted effect of FH on biomarker values. In univariate analyses (Figures 1a–d), mean CSF t-tau/Aβ42 ratios were in general higher in FH+ subjects than in FH− subjects across all diagnostic groups but FH effects were strongest in MCI. Among MCI subjects, as shown in Figures 1 a–c, CSF Aβ42 was lower (p = .005), t-tau was higher (p = 0.02) and t-tau/Aβ42 ratio was higher (p = 0.002) in FH+ than in FH− subjects. Among CN subjects, there was no significant difference for CSF Aβ42 to be lower (p = 0.12) and t-tau/Aβ42 to be higher (p = 0.13) in FH+ than in FH− subjects. Among AD subjects, the effect of FH was also not significant for any comparison (p>0.2 for all). Hippocampal volume (sum of left and right) did not significantly differ by FH status in any group. Additionally, there were no significant differences in ICV between FH+ and FH− in any group (p = 0.51, 0.28, 0.22 for AD, CN, MCI respectively; Figure 2).

### ApoE4 Prevalence and Effects

The ApoE4 allele was overrepresented in FH+ subjects (p = 0.0002) (Table 1) as expected. The % prevalence rate of E4+ was 42% CN, 68% MCI and 75% AD in FH+ subjects versus 21% CN, 42% MCI and 59% AD for FH− subjects, respectively.

### Effect of FH after Adjusting for ApoE4

After adjusting for age, gender, baseline cognition and ApoE4, the effects of FH+ status on CSF t-tau/Aβ42 ratio in MCI subjects remained significant (p<0.036) but showed no significant difference in CN (p = 0.11). As with univariate analyses, the FH effect on t-tau/Aβ42 was again not significant in AD subjects. In these multivariate models, the effect of ApoE4 on t-tau/Aβ42 ratio was significant in CN (p = 0.0082) and MCI (p = 0.000) but not in AD (p = 0.13) (Table 2). The effect of FH on t-tau/Aβ42 ratio in MCI subjects remained significant (p = 0.036) in the model that adjusted for age, gender, E4 and education.

The effect of FH on HV was not significant in any of the three diagnostic groups (Table 3). Age and cognitive status had bigger effects than FH status on HV. The effect of ApoE4 on HV was not significant in CN (p = 0.86), became significant in MCI (p = 0.02) and turned nonsignificant in AD (p = 0.44) (Table 3). ICV did not differ by FH or ApoE4 in any diagnostic group but differed by gender. In another model that adjusted for age, gender, education, E4 and ICV, the effect of FH on HV was not significant in any of the diagnostic groups (p = 0.96, p = 0.66, p = 0.23, respectively) thus adjusting for ICV did not alter the findings.

### Prevalence of “Pathologic” AD Phenotype by FH Status

More than twice as many FH+ CN (47%) exhibited “pathologic phenotype of AD” (CSF t-tau/Aβ42 ratio >0.39) than FH− controls (21%) (p = 0.03). There was also a trend for a greater proportion of FH+ MCI subjects (82%) who exhibited this phenotype than in the FH− MCI group (66%) (p = 0.07). This was not statistically different in the AD group (95% vs 88%, p<0.65).

### ROC Curves

The sensitivity and specificity of CSF t-tau/Aβ42 ratio of 0.39 for separating AD from all CN was 92% and 66%, respectively. For separating AD just from the FH+ CN group sensitivity was 95% and specificity was 53%; for separating AD from the FH− group, sensitivity was 88% and specificity was 79%. The difference in sensitivity between FH+ and FH− groups was not significant (p = 0.65, chi-squared test) while the difference in specificity between FH+ and FH− groups was significant (p = 0.03).

## Discussion

Three main findings emerged from our study. We found significantly higher t-tau/Aβ42 ratios and a higher prevalence of the rate of a “pathologic t-tau/Aβ42 endophenotype” in FH+ (versus FH−) CN and MCI groups. There was an additional effect of FH on these markers above and beyond that of ApoE4 in MCI subjects and the model estimated suggests that this additional effect is about half of the size of the ApoE4 effect. We found no FH effects on CSF pathologic markers in AD. We also found no FH effects on neuronal loss marker (hippocampal volume) both before and after adjusting for intracranial volume. We also found no FH effects on ICV. These data extend findings from prior studies of FH effects [5]–[17] to the national ADNI sample and to MCI subjects. Our study also found that almost half of all normal controls with FH+ would have met research criteria for preclinical AD (based on CSF) [23] at entry into ADNI but only about 20% of FH− subjects would have met such criteria. This result is also consistent with the view that a family history of AD does not reduce cognitive reserve, as there were no significant differences in cognitive test scores between FH+ and FH− groups. Rather, one can speculate that the risk of family history is probably mediated by earlier development of amyloid pathology or more rapid development of amyloid pathology with the same time of onset.

Of prior studies examining FH effects, three are particularly relevant to our analyses. In a study of 269 cognitively normal controls, Xiong et al [12] reported that FH status was linked to a decrease in CSF Aβ42, a finding that we extend by reporting a similar and even more robust change in MCI. Honea et al [11] examined the relationship between biomarkers and parental history of all dementia types in the ADNI sample, thus potentially including also FH of vascular dementia, DLB or FTD or other etiologies. In their study, the rate of FH+ subjects was different from ours and unlike our study, the effects of FH on t-tau/Aβ42 ratio and t-tau effects in MCI did not reach significance. They did report a significant FH effect on Aβ42 in MCI consistent with our finding, but in their study the FH effects in MCI were not significant after adjusting for ApoE4, whereas ours remained significant. They also found pathologic signatures of AD in a smaller percent of CN than we did. Thus, their looser definition appears to have resulted in an underestimation of the effect of FH of AD. Andrawis et al [18] found MCI subjects with positive maternal history of dementia had smaller baseline hippocampal volumes and greater 12-month atrophy rates. The effect of positive maternal history of dementia on hippocampal atrophy in MCI and AD was significant after controlling for age, ApoE4 genotype, and paternal history of dementia. Taken together, these studies along with prior studies showing potential FH effects on brain networks and glucose metabolism highlight the need to further examine FH effects on multiple biomarkers simultaneously.

The mechanisms underlying the effects of FH status are not fully known but will likely vary depending on biomarker – ie genetic mechanisms underlying hippocampal volume changes are not likely to be identical to those underlying amyloid or tau processing. Prior autopsy, CSF and PET studies have linked ApoE4 to an amyloid phenotype and hippocampal changes [6]–[10] [26], and studies have documented ApoE4 effects on greater neocortical amyloid-beta deposition and/or reduced clearance [6] [27]. However, E4 does not account for all of the variance and there is interest in determining the degree of unexplained heritability not accounted for by ApoE4 as well as the genes underlying such unexplained heritability. In our ADNI sample, the effect of adjusting for ApoE4 on pathologic markers was different in different diagnostic groups - the FH effect was considerably weakened in CN, remained significant in MCI subjects, and was not significant in AD. After adjusting for ApoE4, the remaining FH effect on CSF t-tau/Aβ42 was approximately half the size of the main ApoE4 effect. Thus, our data along with others [8] [12] confirms that there are as yet unidentified genetic factors embedded in FH status that influence pathology before the onset of dementia [12].

Our sensitivity/specificity analyses also suggest that the presence of FH+ controls in an AD control group may significantly reduce the specificity of CSF pathologic biomarkers for separating AD from controls. It may be worth examining whether including FH+ controls may have reduced the accuracy of calculations in other tests, such as amyloid PET or plasma Aβ42. Likewise our data also suggests that companies planning registration studies of diagnostic biomarkers to detect AD pathology in at-risk subjects may wish to exclude FH+ controls to enhance their power for achieving the desired 80% or greater specificity.

There are also some potential limitations of this study – by design ADNI’s sample size of controls and AD was relatively smaller than the MCI group, which may have limited power to detect small effects in controls. CSF data were only collected in a subset who agreed to volunteer, a selection bias that applies to most CSF biomarker studies. FH status was determined through interviews with subjects and informants, but may have been subject to a reporter bias and lack of informative pedigree (early death of relatives due to other causes, though this problem is less likely in the US due to longer life expectancies and greater awareness of dementia). Many respondents may not be well versed enough to know the difference between a dementia and AD. Because FH in most biomarker research and practice is usually collected only by simple history, our findings are relevant. We also did not distinguish between maternal and paternal inheritance and hence our data cannot be compared with findings that maternal family history may have greater risk for metabolic changes or hippocampal atrophy [15]–[16]. Furthermore, given that there is a mitochondrial hypothesis providing an underlying biological mechanism for promoting disease on the maternal side we believe future studies should examine maternal versus paternal family history. However a recent longitudinal study of 108 middle-aged normal controls (of younger age than ADNI cohort) found that FH status predicted greater atrophy only within a posterior sub-region of the hippocampus but not in other gray matter regions, and that there was no effect of maternal versus paternal history [26]. Differences in sampling, FH ascertainment, and biomarker methods could account for some of the discrepant findings. While the means differ significantly, the overlap in CSF data boxplots between FH+ and FH− MCI groups suggests that these findings may not be as robust a biomarker as one where the boxplots do not overlap at all - unfortunately no such biomarker exists.

What do these phenotypic differences related to a positive FH in MCI mean for the subject? Other studies have linked CSF pathologic phenotypes with faster rates of future decline in CN and MCI subjects [22]. By extrapolation, this would imply that the subset of FH+ MCI and CN subjects with abnormal biomarker phenotypes would decline faster than FH− subjects. Longitudinal data from ADNI and standardization of hippocampal sub-region analyses as well as CSF soluble amyloid oligomer assays [28] may permit more definitive testing of the prognostic significance of FH differences on risk for decline.

In summary, our study, derived from a large national biomarker cohort, documents that a positive family history of AD is associated with an abnormal beta-amyloid and tau endophenotype prior to the onset of clinical AD in mildly symptomatic subjects, and that there are genetic influences embedded within FH beyond that due to ApoE4 that are most obvious in the MCI cohort. Since CSF biomarkers correlate highly with cerebral neuritic beta-amyloid and neurofibrillary tangle changes [28], we also speculate that FH status is associated with earlier onset of preclinical pathologic AD. These findings have implications for the design of future research studies, heritability of AD and personalizing testing and care of at risk subjects.
